# Supplementary material for: The Kinase Chemogenomic Set (KCGS): An Open Science Resource for Kinase Vulnerability Identification
Source: Int J Mol Sci. 2021 Jan 8;22(2):566. doi: 10.3390/ijms22020566 (PMC7826789; doi:10.3390/ijms22020566)
Supplement: Supplementary file 1 [file ijms-22-00566-s001.zip › SI 2 Protocol for dilution and use of KCGS.pdf]

Protocol for dilution and use of KCGS

96-well plate assay single concentration screen at 1 μM

Step 1: Add to each well of the KCGS plate 9 μL of DMSO (initial concentration 10 mM; final concentration 1 mM)

Step 2: Take 1 μL from each well and transfer to a daughter plate in the desired screening format

Step 3: Add 9 μL of media to each well of the daughter plate (initial concentration 1 mM; final concentration 100 μM)

Step 4: Add 1 μL of the liquid from Step 3 to the cell-based assay (100 μL total assay volume) to generate a final concentration of 1 μM of the inhibitor with a DMSO concentration of 0.1%.

These steps provide enough volume to perform an assay allowing for both technical and biological replicates as well as a counter screen in a control cell line.
